# MiR-138-5p targets RUNX2 to inhibit osteogenic differentiation of aortic valve interstitial cells via Wnt/β-catenin signaling pathway

**DOI:** 10.1186/s12872-022-02471-6

**Published:** 2022-02-02

**Authors:** Fei Yan, Qiang Huo, Weimin Zhang, Tingting Wu, Daniyaer Dilimulati, Lin Shi

**Affiliations:** 1 Department of Cardiac Surgery, The First Affiliated Hospital of Xinjiang Medical University, 137 Liyushan South Road, Xinshi District, Urumqi, 830054 Xinjiang China; 2 Department of Cardiology, The First Affiliated Hospital of Xinjiang Medical University, 137 Liyushan South Road, Xinshi District, Urumqi, 830054 Xinjiang China

**Keywords:** Calcific aortic valve disease (CAVD), Aortic valve interstitial cells (AVICs), Osteogenic differentiation, miR-138-5p, RUNX2, Wnt/β-catenin

## Abstract

**Background:**

Human aortic valve interstitial cells (hAVICs) are a key factor in the pathogenesis of calcific aortic valve disease (CAVD). This research examines the role and mechanism of microRNA miR-138-5p in osteogenic differentiation of hAVICs.

**Methods:**

RT-qPCR analysis was applied for detecting miR-138-5p and RUNX2 expression in valve tissues of CAVD patients and controls. On completion of induction of osteogenic differentiation of hAVICs, and after overexpression or interference of miR-138-5p expression, the condition of osteogenic differentiation and calcification of hAVICs was confirmed using alkaline phosphatase staining and alizarin red staining. Subsequently, western blot was utilized to detect the expression of osteogenesis-related proteins OPN and ALP, and Wnt/β-catenin signaling pathway-related proteins. Finally, the relationship between miR-138-5p and RUNX2 was validated by dual-luciferase reporter assay and Pearson’s correlation test.

**Results:**

Down-regulation of miR-138-5p was found in CAVD patients and during osteogenic differentiation of hAVICs. Overexpression of miR-138-5p contribute to the inhibition of osteoblast differentiation and calcium deposition in hAVICs, and of ALP and OPN protein expression. RUNX2 was a target gene of miR-138-5p, and it was negatively correlated with miR-138-5p in CAVD. Additionally, overexpression of RUNX2 could reverse the inhibitory effect of miR-138-5p on osteogenic differentiation of hAVICs.

**Conclusion:**

miR-138-5p can act as a positive regulator of osteogenic differentiation in CAVD patients to involve in inhibiting valve calcification, which is achieved through RUNX2 and Wnt/β-catenin signaling pathway.

**Supplementary Information:**

The online version contains supplementary material available at 10.1186/s12872-022-02471-6.

## Introduction

Calcific aortic valve disease (CAVD), with the prevalence ranking first among valvular heart diseases clinically, is characterized by valve thickening and calcification, aortic hemodynamic disorders and heart failure, which is mainly responsible for valve replacement failure. Among the numerous etiological factors of CAVD, the osteogenic differentiation of aortic valve interstitial cells (AVICs) that mainly regulate aortic valve structure and function, and subsequent calcium deposition are considered to be key factors [1–4].

MicroRNAs (miRNAs) refer to small non-coding RNA molecules which are highly conserved and single-stranded. They mediate the levels of their target genes through transcriptional repression or mRNA degradation, thus regulating a variety of physiological and pathological processes [5], including cell proliferation, apoptosis [6], inflammation [7], and cancer [8]. In recent years, many miRNAs involved in osteogenic differentiation of AVICs have been identified. For example, miR-29b activates wnt3/β-catenin/Smad3 signaling to inhibit TGF-β3 and consequently to promote AVICs calcification [9], while miR-638 targets Sp7 to inhibit human AVICs (hAVICs) calcification by targeting Sp7 [10]. Among miRNAs, regulation of miR-138-5p in various biological processes has been proved, such as its involvement in developmental events associated with cell differentiation. For example, osteogenic differentiation of human mesenchymal stem cells (hMSCs) can be negatively regulated by miR-138-5p; this miRNA mediates AK-ERK1/2 activity, thereby decreasing Runx2 expression and achieving the negative regulation [11]. By contrast, this process can be promoted by miR-138-5p knockdown and the resulting up-regulation of FOXC1 [12]. However, what and how miR-138-5p affects CAVD has not been reported. Therefore, this study first clarifies miR-138-5p expression during osteogenic differentiation of hAVICs, and then its specific function and mechanism of miR-138-5p in this process are confirmed by cell experiments. The findings of this research provide a potential strategy for the prevention or treatment of multiple valvular calcification-associated diseases.

## Materials and methods

### Collection of human aortic valve tissue

Human aortic valve tissue (n = 10) were obtained from calcified aortic valves of adult patients undergoing aortic valve replacement in our hospital, and non-calcified aortic valves of patients with aortic annular dilatation-caused aortic regurgitation (n = 5). Pathomorphologic diagnosis were confirmed at the time of aortic valve replacement. Patients with a history of infective endocarditis or rheumatic heart disease were excluded. All patients had signed informed consent. The study was approved by the ethics review board of our hospital and conformed to the principles specified in the Declaration of Helsinki, with an approval by the Ethics Committee of The First Affiliated Hospital of Xinjiang Medical University (K202107-09).

### Isolation and culture of hAVICs

hAVICs were isolated as described by Zhang et al. [2]. First, on completion of aseptic acquisition of the aortic valve from calcified aortic valves and normal controls, endothelium and non-lobular tissue were removed from the ventricles and aorta. Subsequently, the lobule was soaked in 0.25% trypsin for 5 min and then cut into 3 × 3 mm pieces. Next, after 12 h digestion of the pieces by trypsin at ambient temperature, the digested cells were collected and transferred to DMEM medium (Sigma, USA). The medium containing l-glutamine, 10% fetal bovine serum (FBS), 10 U/L penicillin, and 10 μg/L streptomycin was placed in an incubator (5% CO_2_, 37 °C), and changed every 3 days. The cells were examined microscopically for purity after five passages.

### Transfection of hAVICs

hAVICs were plated in 6-well plates with a density of 5 × 10^4^ cells/well until reaching 60–70% confluence. After that, 2 nM miR-138-5p mimics, miR-138-5b inhibitor or control were transfected into cells using Lipofectamine2000 transfection kit (Invitrogen), respectively. Then with low serum medium (contained 1% FBS, changed every 3 days) for culturing the transfected cells, cells were collected after 21 days for subsequent analysis.

### Osteogenic differentiation induction of hAVICs

hAVICs were plated in 24-well plates with 4 × 10^4^ cells in each well until reaching 60–70% confluence. After that, osteoblastic differentiation was induced by continuous culture in medium containing 50 mg/mL ascorbate-2-phosphate and 10 mM β-glycerophosphate for 21 days, with the culture in blank medium as a control. On the 7th day and 21st day of induction, alkaline phosphatase (ALP) staining and alizarin red staining were performed to evaluate osteoblast induction.

### Alizarin red staining

Primary hAVICs cultured in medium containing 10 mM β-glycerophosphate were fixed in 70% ethanol for 1 h at ambient temperature and then stained with 40 mM alizarin red (Beyotime, China) for 10 min. On completion staining, cells were rinsed with PBS to clear nonspecific staining, and the stained matrix was photographed using a digital microscope. Afterwards, the alizarin red S dye was released from the cell matrix by incubation in cetyl pyridinium chloride for 15 min, followed by staining quantification of the amount of the released dye by spectrophotometry at 540 nm. Finally, the quantitative results were normalized to the total cellular protein content [13].

### Alkaline phosphatase (ALP) staining

After osteogenic induction of hAVICs and removal of the original medium, the cells were rinsed by PBS, and then stained with an appropriate amount of BCOP/NBT staining solution (Thermo, USA) for 25 min at room temperature in the dark. On completion staining, cells were rinsed with PBS to clear nonspecific staining, and the stained matrix was photographed using a digital microscope. Afterwards, 400 µL of cell lysate was added to each well, and finally the cell supernatant was collected after centrifugation (1000 g, 10 min).

### Detection of alkaline phosphatase (ALP) activity

Detection of ALP activity of hAVICs was completed by utilizing an alkaline phosphatase activity colorimetric assay kit (BioVisionInc., Mountain View, CA). On completion of rinsing step by PBS, transfected cells were added with 50 ml of buffer to homogenize. After that, the insoluble materials were removed through centrifugation (13,000 g, 3 min), and the samples were plated to a 96-well plate in equal volumes at different concentrations. Then, with 5 mM of 50 mol/l pNPP solution in each control well and test well, an incubation for a period of 60 min was performed at ambient temperature in the dark. Finally, based on the standard curve, ALP activity was calculated: ALP activity (U/mL) = A (amount of pNP produced by each sample, μmol)/V (volume of the reaction system, mL)/T (time of reaction, min).

### RT-qPCR

TRizol reagent (Invitrogen, USA) was adopted to extract the total RNA from hAVICs, and Toyoba Reverse transcription kit (Fermentas, Canada) for reverse transcription from RNA to cDNA. Subsequently, ABI PRISM7900 Sequence Detection System (Applied Biosystems, Foster City, CA) was employed in RT-qPCR. Ultimately, with GAPDH and U6 as internal according to the instructions. GAPDH gene and U6 were used as reference genes, relative quantification of target gene expression was completed by 2^−ΔΔCt^ method (Table 1). Three independent experiments were in 3 replicates.

**Table 1:** Primer sequences

Gene	Sequence (5′–3′)
miR-138-5p	F AGCTGGTGTTGTGAATCAGGCCG
	R AACGCTTCACGAATTTGCGT
U6	F CTC GCTTCGGCAGCACA
	R AACGCTTCACGA ATTTGCGT
RUNX2	F ATGGCATCAAACAGCCTCTTCAGC
	R TCAATATGGTCGCCAAACAGATTCA
GAPDH	F ATGGTTTACATGTTCCAATATGA
	R TTACTCCTTGGAGGCCATGTGG

### Dual-luciferase reporter assay

The binding site of miR-138-5p and RUNX2 gene was predicted utilizing TargetScan database (http://www.targetscan.org/vert_72/). On the completion of the construction of RUNX2-WT and RUNX2-MUT vectors, 293T cells cultured in 24-well plates were transfected with these vector and miR-138-5p mimics by using Lipofectamine2000 (Invitrogen), respectively. The cells were collected 48 h after transfection, and lysed using lysate (Beyotime, China), followed by measurement of luciferase activity with the dual luciferase reporter assay system (Promega). Each group is provided with two parallel wells, and the experiment was in 3 replicates.

### Western blot

Total protein was extracted from hAVICs using the Radio immunoprecipitation assay (RIPA), and the protein concentration was determined by a BCA kit (Thermo, USA). Then after separation using 10% SDS-PAGE, the proteins were blotted onto PVDF membranes at 200 mA, followed by 1 h blocking with 5% skimmed milk at room temperature. Subsequently, the membranes were incubated with antibodies against RUNX2, Wnt5a, β-catenin, OPN, ALP and β-actin antibodies overnight at 4 °C. After washing with PBST for 3 times, the membranes were then incubated with secondary antibodies at room temperature for 2 h and rinsed with PBST for three times again. Finally, an exposure apparatus was used to develop proteins, and ImageJ software for grayscale analysis. Relative protein expression was calculated using β-actin as a control.

### Statistical analysis

SPSS 21.0 software was utilized for statistical analysis, and data were expressed as mean ± standard deviation (SD). Student’s *t*-test was performed for two groups comparisons. One-way analysis of variance (ANOVA) followed by multiple comparisons was carried out. A statistically significant difference can be suggested if *P* < 0.05.

## Results

### miR-138-5p affects osteogenic differentiation of hAVICs

**Fig. 1:**
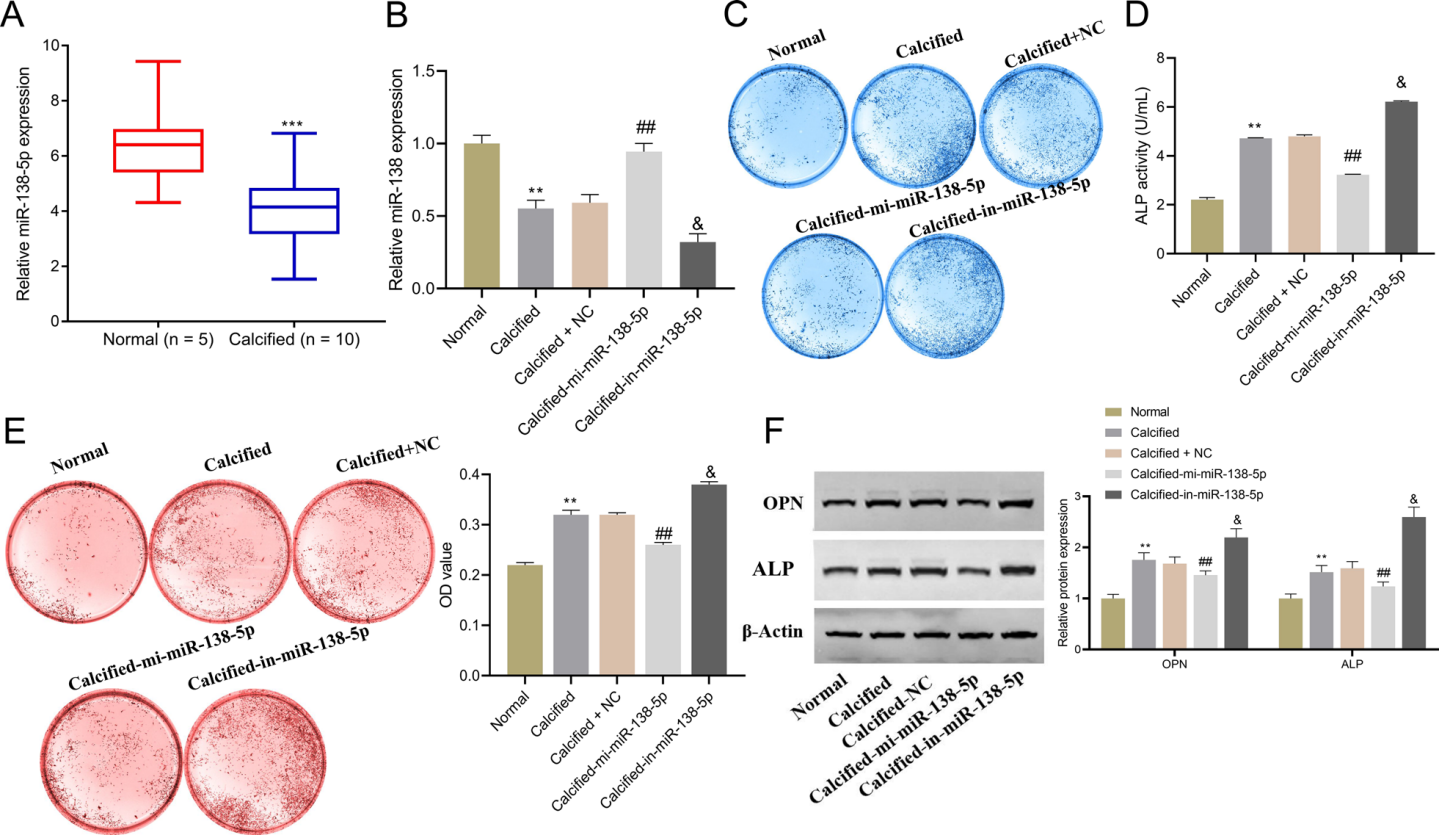
miR-138-5p expression affects the osteogenic differentiation of human aortic valve interstitial cells (hAVICs). **A**, **B** RT-qPCR-based detection of miR-138-5p expression in valve tissues of CAVD patients (calcified aortic valves) and normal controls (normal aortic valves) (**A**), and in human aortic valve interstitial cells (hAVICs) (**B**); ***P < 0.001 versus Normal group. **C** ALP staining for evaluating osteogenesis of hAVICs; **D** ALP activity of hAVICs; **E** Alizarin red staining for detecting calcification of hAVICs; **F** Assessment of OPN and ALP protein expression by western blot and quantitative analysis; **P < 0.01 and ***P < 0.001 versus Normal; ^##^*P* < 0.01 versus Calcified group; &*P* < 0.05 versus Calcified-NC group

First, by using RT-qPCR for determining miR-138-5p expression in the valve tissues of CAVD patients, significantly lower miR-138-5p was identified in the CAVD patients compared with the controls (Fig. 1A). Further, cell experiments confirmed what effects miR-138-5p had on osteogenic differentiation and calcification of hAVICs. As shown in Fig. 1B, miR-138-5p was lowly expressed during osteogenic differentiation of hAVICs. Up-regulation of miR-138-5p expression by transfection of miR-138-5p mimics and down-regulation of its expression by miR-138-5p inhibitor, suggested successful transfection (Fig. 1B). In addition, overexpression of miR-138-5p was responsible for the inhibition of ALP activity and calcification in hAVICs (Fig. 1C–E), and expression of osteogenic differentiation markers (osteopontin (OPN) and ALP proteins) (Fig. 1F). By contrast, inhibiting miR-138-5p could achieve opposite results. Collectively, miR-138-5p was downregulated in osteogenic differentiation of hAVICs, while overexpressed miR-138-5p could significantly inhibit osteogenic differentiation and calcification of hAVICs.

### MiR-138-5p targets RUNX2 in CAVD

**Fig. 2:**
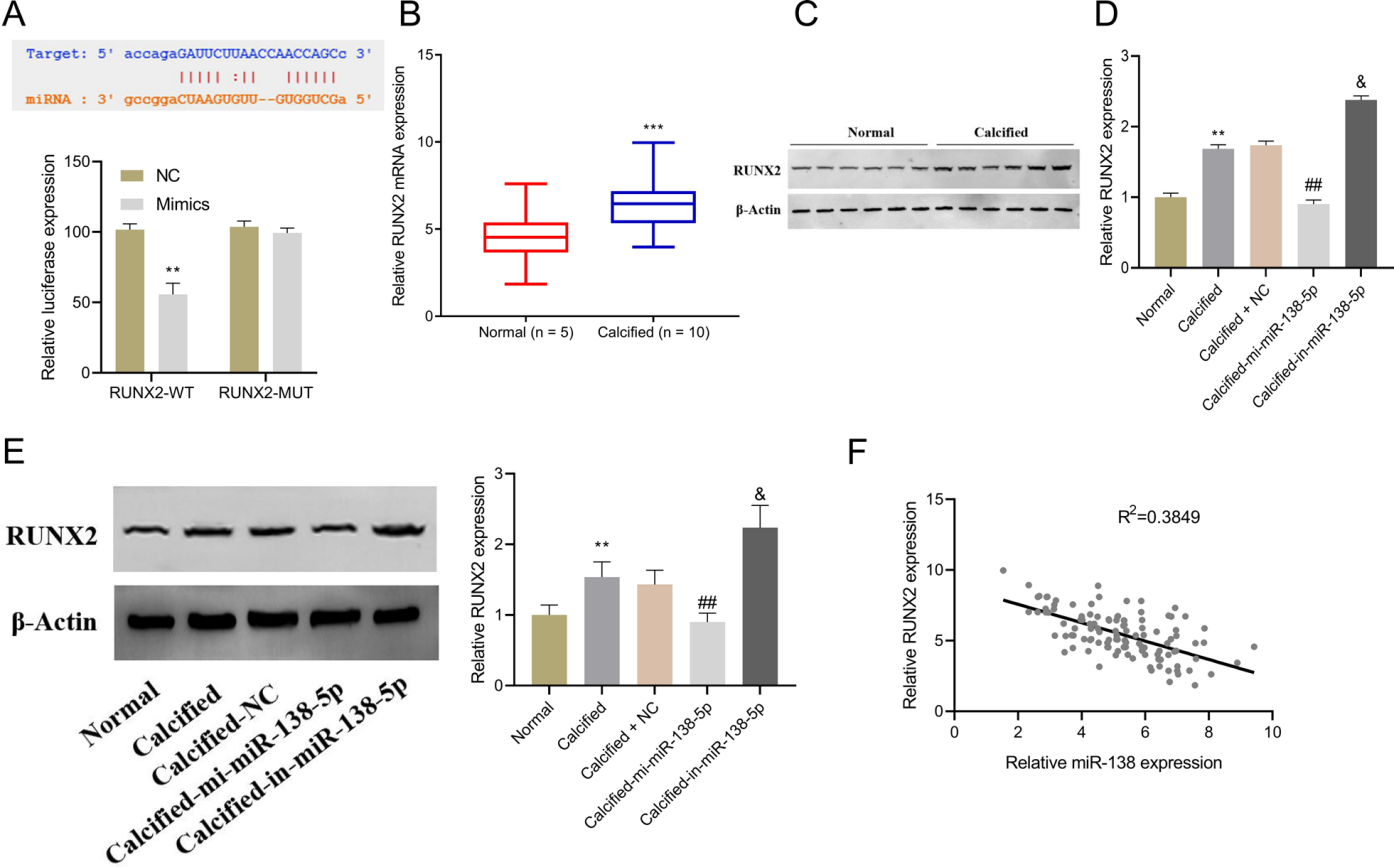
RUNX2 is a direct target of miR-138-5p. **A** Prediction of the target gene for miR-138-5p by TargetScan, and subsequently validation by dual-luciferase reporter assay; **B**, **C** RT-qPCR-based detection (**B**) and western blot-based assessment (**C**) of RUNX2 expression in valve tissues of CAVD patients (calcified aortic valves) and controls (normal aortic valves); ***P < 0.001 versus Normal group. **D**, **E** RT-qPCR detection (**D**) and western blot assessment (**E**) of RUNX2 expression in human aortic valve interstitial cells; **F** Pearson analysis of RUNX2 and miR-138-5p expression. **P < 0.01 versus normal group; ^##^*P* < 0.01 versus Calcified group; &*P* < 0.05 versus Calcified-NC group

How miR-138-5p inhibited osteogenic differentiation of hAVICs was further investigated. TargetScan database predicted the binding site between RUNX2 and miR-138-5p, and the prediction was confirmed by dual-luciferase reporter assay (Fig. 2A). In comparison with the controls, significant up-regulation of both RUNX2 mRNA and protein was found in the valve tissues of CAVD patients (Fig. 2B, C). During osteogenic differentiation of hAVICs, overexpressed miR-138-5p inhibited RUNX2 mRNA and protein expression, while miR-138-5p knockdown achieved an opposite result (Fig. 2D, E). Further correlation analysis indicated a negative correlation between RUNX2 and miR-138-5p expression in CAVD (Fig. 2F).

### MiR-138-5p affects osteogenic differentiation of hAVICs by targeting RUNX2

**Fig. 3:**
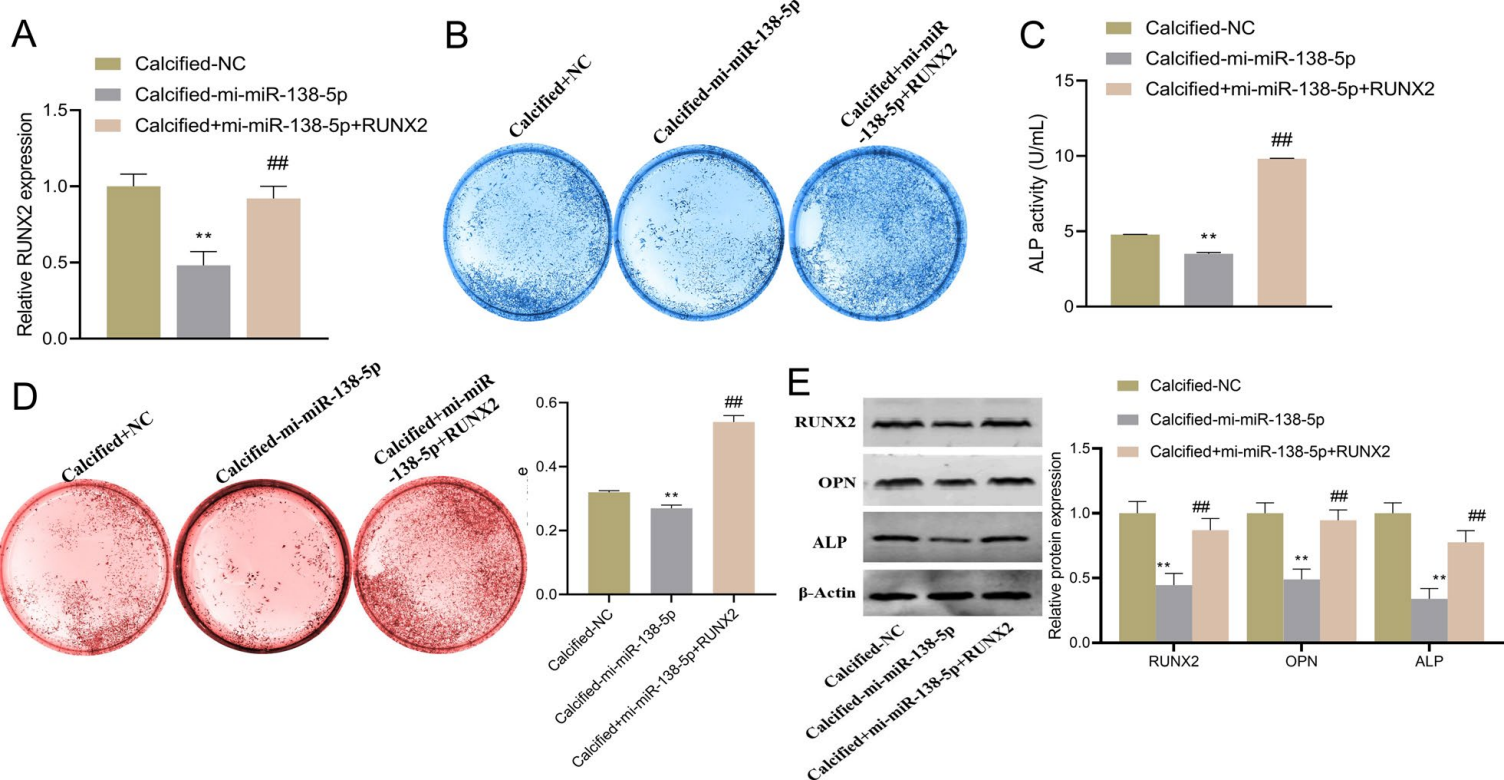
MiR-138-5p inhibits osteogenic differentiation of hAVICs by decreasing RUNX2. **A** RT-qPCR for detecting RUNX2 expression in hAVICs; **B** Evaluation of osteogenesis of hAVICs by ALP staining; **C** ALP activity of hAVICs; **D** Alizarin red staining for confirming calcification of hAVICs; **E** Western blot-based detection of RUNX2, OPN and ALP proteins in hAVICs. **P < 0.01 versus Calcified-NC group; ^##^*P* < 0.01 versus Calcified-mi-miR-138-5p group

Whether RUNX2 involved in the mechanism of miR-138-5p affecting osteogenic differentiation of hAVICs was investigated by cell rescue assays. Specifically, in comparison with Calcified-NC group, RUNX2 expression in the Calcified-mi-miR-138-5p group was significantly lower, while an increase of RUNX2 expression was found in the Calcified + mi-miR-138-5p + RUNX2 group (Fig. 3A). Overexpressed RUNX2 relieved the inhibitory effect of up-regulation of miR-138-5p on ALP activity and calcification in hAVICs (Fig. 3B–D), while it increased OPN and ALP protein expression (Fig. 3E). Taken together, the inhibitory effect of overexpressed miR-138-5p on osteogenic differentiation was achieved by decreasing RUNX2.

### MiR-138-5p regulates RUNX2-Wnt/β-catenin axis to inhibit osteogenic differentiation of hAVICs

**Fig. 4:**
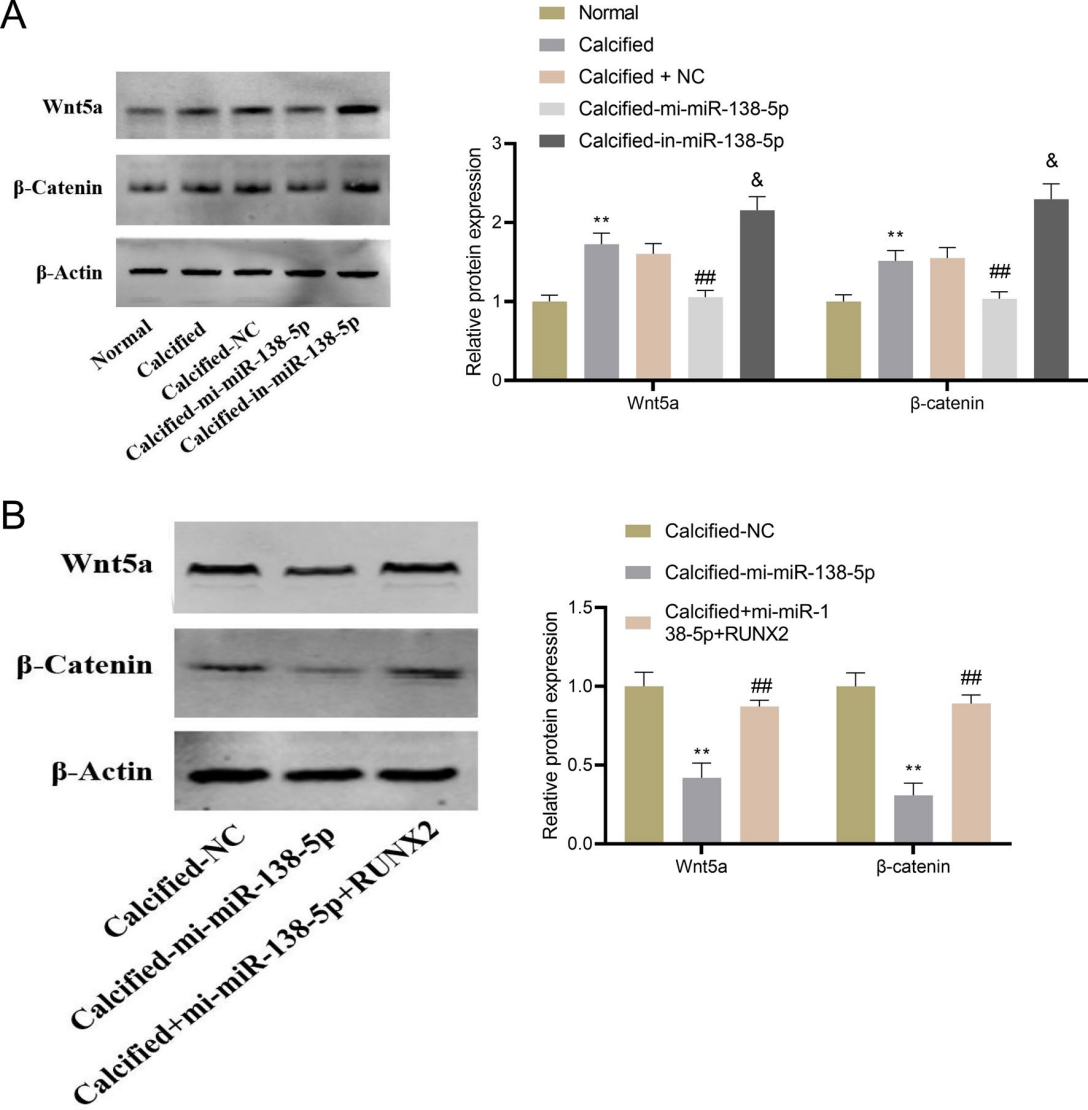
Effect of miR-138-5p targeting RUNX2 on Wnt/β-catenin signaling pathway. **A** Western blot-based detection of Wnt5a and β-catenin protein expression in osteogenically differentiated hAVICs transfected with miR-138-5p mimics or inhibitor. **P < 0.01 versus Normal group; ^##^*P* < 0.01 versus Calcified group; &*P* < 0.05 versus Calcified-NC group. **B** Western blot-based detection of Wnt5a and β-catenin protein expression in osteogenically differentiated hAVICs transfected with miR-138–5 mimics or miR-138-5p mimics combined with RUNX2 overexpression plasmid

Involvement of Wnt/β-catenin signaling pathway in osteogenic differentiation has been proved [14]. For further investigation of the mechanism by which miR-138-5p inhibited osteogenic differentiation of hAVICs, Wnt/β-catenin signaling pathway-related proteins were detected. According to the results, overexpression of miR-138-5p contributed to reduce Wnt5a and β-catenin protein expression, while miR-138-5p knockdown achieved the opposite outcomes (Fig. 4A). Simultaneous overexpression of RUNX2 and miR-138-5p could reversed the inhibitory effect of overexpressed miR-138-5p on Wnt5a and β-catenin (Fig. 4B). The above results illustrated that the inhibitory effect of miR-138-5p on osteogenic differentiation was dependent on the Wnt/β-catenin signaling pathway.

## Discussion

CAVD is one of the main causes of death and morbidity of cardiovascular diseases, especially in the elderly, and development of this disease is associated with osteogenic differentiation (or calcification). However, there are no specific drugs effective for CAVD from lack of insight into the pathomechanism of osteogenic differentiation. Aortic valve replacement is currently the only means to alleviate the condition. But in recent years, it has been reported that osteogenic differentiation also occurs in the surgically implanted aortic valves, and AVIC, the main cell of aortic valve tissue, is also involved in this pathological process. Therefore, for treating CAVD, there is a need for an investigation on the mechanism of aortic valve calcification [15]. In this study, we found that miR-138-5p negatively regulates the process of osteogenic differentiation of human calcified aortic valve by targeting RUNX2.

Among miRNAs, miR-138-5p has gradually received attention in the regulation of osteogenic differentiation. For example, hsa-miR-138 inhibits adipogenic differentiation of adipose tissue-derived hMSCs through adenovirus EID-1 [16], and down-regulation of Noggin and miR-138 synergistically promote osteogenic transformation of MSCs [17]. In our study, miR-138-5p was down-regulated in CAVD patients, and was down-regulated during osteogenic differentiation of hAVICs. OPN, a highly phosphorylated glycoprotein secreted by osteoblasts, can be deposited into the bone matrix to promote osteocyte adhesion; OPN is one of the phenotypes of osteoblasts, and participates in resorption, mineralization of bone matrix and in maintenance of bone tissue integrity [18]. ALP, a marker of osteoblast, can be adopted for detecting the degree of differentiation of osteoblasts [19]. In this study, we also found that up-regulation of miR-138-5p inhibited OPN and ALP protein expression and ALP activity, and at the same time, and ALP staining and alizarin red staining also indicated that overexpressed miR-138-5p inhibited osteogenic differentiation of hAVICs.

RUNX2 is a core molecule in osteogenic differentiation [20–23], which can be down-regulated by miR-204-5p to inhibit osteogenic differentiation in rat BMSC. According to the results in this study, both protein and transcript levels of RUNX2 were significantly increased in hAVICs from patients with calcified aortic valve and presented a negative correlation with miR-138-5p. RUNX2 was also confirmed to be a target gene of miR-138-5p, and the inhibitory effect of miR-138-5p on osteogenic differentiation of hAVICs was achieved through RUNX2.

Wnt/β-catenin pathway is reported to be one of the essential pathways for the osteogenic process [14]. Activation of Wnt promotes AVICs to express β-catenin and resulting promotion of transcription and expression of RUNX2 and ultimately osteogenic differentiation of progenitor cells and calcification of vascular smooth muscle cells [24, 25]. However, the relationship between miR-138-5p and Wnt/β-catenin pathway in CAVD is not clear. It has been demonstrated that protein levels of canonical Wnt/β-catenin signaling pathway are markedly changed in human patients, rodent models, and in vitro valve interstitial cells [15, 26, 27]. In the present study, overexpression of miR-138-5p significantly inhibited the expression of Wnt/β-catenin signaling pathway-associated proteins, while simultaneous overexpression of miR-138-5p and RUNX2 reversed this inhibitory effect, suggesting that the Wnt/β-catenin signaling pathway was involved in the regulation of miR-138-5p/RUNX2 on osteogenic differentiation.

## Conclusion

In summary, through RUNX2 and Wnt/β-catenin signaling pathway, miR-138-5p acts as a positive regulator of osteogenic differentiation in CAVD patients to involve in inhibiting valve calcification. This miRNA may become a new marker of osteogenic differentiation, contributing to a potential therapeutic strategy for preventing or treating to diseases associated with valve calcification.

## Supplementary Information

**Additional file 1.** All uncut Western blot bands in the article.

## Data Availability

The data used to support the findings of this study are available from the corresponding author upon request.
