# Supplementary figures and images for: MiR-138-5p targets RUNX2 to inhibit osteogenic differentiation of aortic valve interstitial cells via Wnt/β-catenin signaling pathway
Source: BMC Cardiovasc Disord. 2022 Feb 2;22:24. doi: 10.1186/s12872-022-02471-6 (PMC8811996; doi:10.1186/s12872-022-02471-6)

Figure 1F

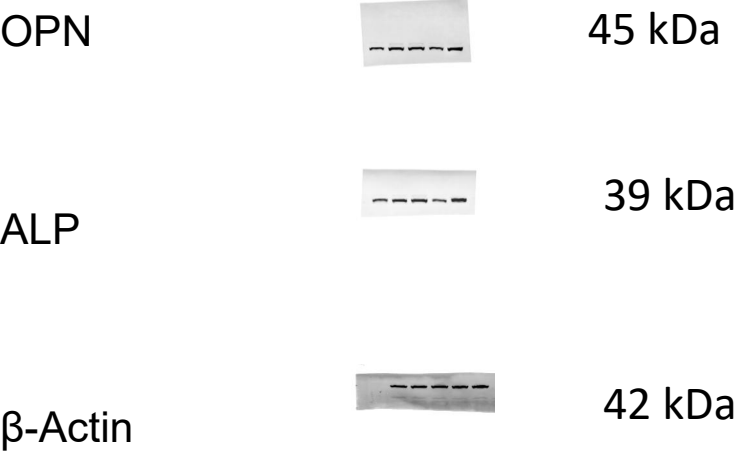

Figure 2C

RUNX2

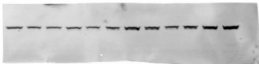

57 kDa

β-Actin

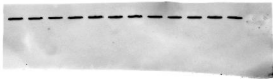

42 kDa

Figure 2D

RUNX2

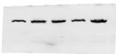

57 kDa

β-Actin

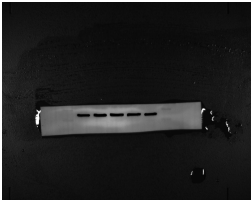

42 kDa

Figure 3E

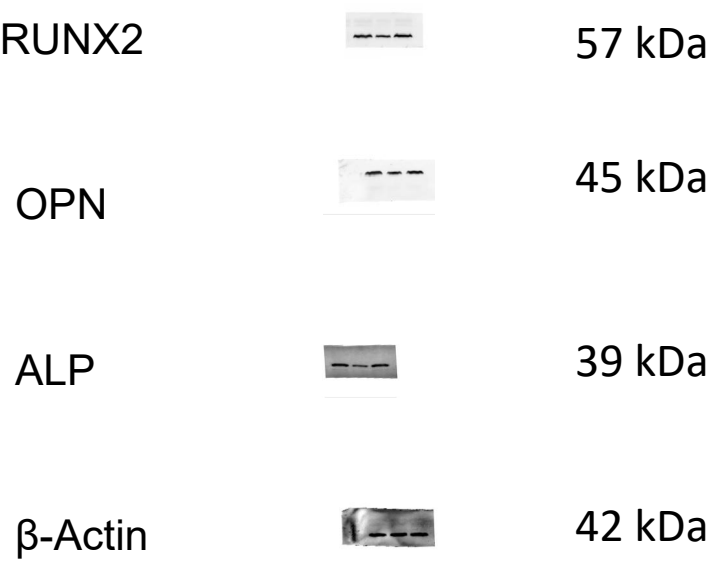

Figure 4A

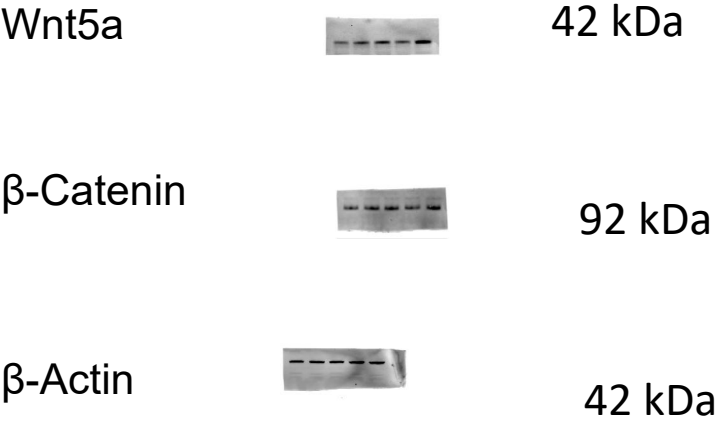

Figure 4B

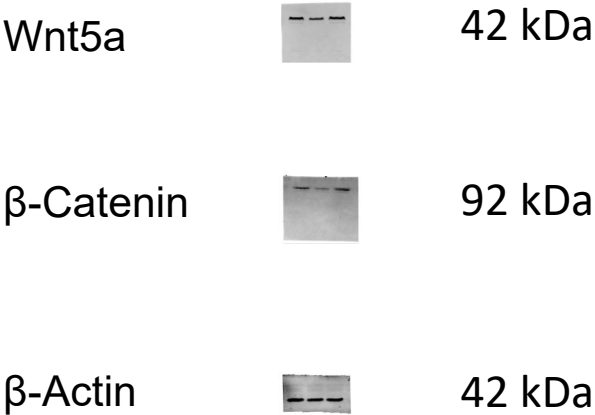

Supplement: Supplementary file 1 — Additional file 1. All uncut Western blot bands in the article. [file 12872_2022_2471_MOESM1_ESM.pdf]
